# Novel in vivo endometriotic models associated eutopic endometrium by implanting menstrual blood-derived stromal cells from patients with endometriosis

**DOI:** 10.1038/s41598-023-35373-4

**Published:** 2023-05-23

**Authors:** Yuejian Zhang, Tiantian He, Taoxiu Lin, Qi Guo, Chaoyue Huo, Song Ze Roberts, Mengping Yang, Sichen Yang, Luyi Gao, Wenjuan Zhang, Changxiang Li, Xiaona Ma

**Affiliations:** 1 The Third School of Clinical Medicine, Beijing University of Chinese Medicine, Beijing, China; 2 Department of Galactophore, Beijing University of Chinese Medicine Affiliated Third Hospital, Beijing, China; 3 The School of Traditional Chinese Medicine, Beijing University of Chinese Medicine, No. 11. Beisanhuang Dong Street, Chaoyang District, Beijing, 100029 China; 4 Department of Gynecology, Beijing University of Chinese Medicine Affiliated Third Hospital, No. 51. Xiaoguan Street, Chaoyang District, Beijing, 100029 China

**Keywords:** Urogenital models, Mesenchymal migration, Endocrine reproductive disorders, Mesenchymal stem cells

## Abstract

The eutopic endometrium provides novel insights into endometriotic pathophysiology and treatment. However, no in vivo models currently available are suitable for eutopic endometrium in endometriosis. In this study, we present new endometriotic in vivo models associated with eutopic endometrium using menstrual blood-derived stromal cells (MenSCs). First, we isolated endometriotic MenSCs (E-MenSCs) and healthy MenSCs (H-MenSCs) from the menstrual blood of patients with endometriosis (*n* = 6) and healthy volunteers (*n* = 6). Then, we identified MenSCs’ endometrial stromal cell properties using adipogenic and osteogenic differentiation. A cell counting kit-8 and wound healing assay were used to compare the proliferation and migration capability between E-MenSCs and H-MenSCs. Seventy female nude mice were used to prepare endometriotic models related to eutopic endometrium by implanting E-MenSCs relying on three approaches, including surgical implantation using scaffolds seeded with MenSCs, and subcutaneous injection of MenSCs in the abdomen and the back (*n* = 10). H-MenSCs or scaffolds only were implanted in control groups (*n* = 10). One month after the surgical implantation and 1 week after the subcutaneous injection, we evaluated modeling by hematoxylin–eosin (H&E) and immunofluorescent staining of human leukocyte antigen α (HLAA). Fibroblast morphology, lipid droplets, and calcium nodules in E-MenSCs and H-MenSCs identified their endometrial stromal cell properties. We noticed that the proliferation and migration of E-MenSCs were considerably enhanced compared to H-MenSCs (*P* < 0.05). E-MenSCs implanted in nude mice formed ectopic lesions using three approaches (*n* = 10; lesions formation rate: 90%, 115%, and 80%; average volumes: 123.60, 27.37, and 29.56 mm^3^), while H-MenSCs in the nude mice shaped nothing at the implantation sites. Endometrial glands, stroma, and HLAA expression in these lesions further verified the success and applicability of the proposed endometriotic modeling. Findings provide in vitro and in vivo models and paired controls associated with eutopic endometrium in women with endometriosis using E-MenSCs and H-MenSCs. The approach of subcutaneous injection of MenSCs in the abdomen is highlighted due to non-invasive, simple, and safe steps, a short modeling period (1 week), and an excellent modeling success rate (115%), which could improve the repeats and success of endometriotic nude mice model and shorten the modeling period. These novel models could nearly intimate human eutopic endometrial mesenchymal stromal cells in the progress of endometriosis, opening a new path for disease pathology and treatment.

## Introduction

Endometriosis is a common gynecologic disorder that affects 20–50% of women with infertility, 71–87% of patients with chronic pelvic pain in China^1^, and 25–75% of dysmenorrhea in teenagers around the globe^2^. The latest review about this condition^3^ summarized 176 million women of reproductive age with endometriosis worldwide and presented data that showed higher incidence rates in younger people. Despite its widespread presence, prolonged diagnosis, and misdiagnosis of endometriosis are still common^4^, so effective therapies are often delayed, affecting the quality of life of women suffering from the condition. Furthermore, the pathology of endometriosis has not been clarified, and the medical community still relies on hypotheses such as retrograde menstruation, coelomic metaplasia, and Müllerian remnants^5^. Endometriosis is now considered a systematic disease that affects far beyond the pelvis—metabolism, systemic inflammation, and gene expressions are all influenced by the condition^6^.

Animal models of endometriosis appeared as an essential adjunct to gain insights into this disorder's etiology and pathophysiological mechanisms. However, surgical endometrial transplantation to other tissues of syngeneic animals^7–9^, which is now the classical endometriosis modeling, is not effective to report the human endometrial properties that are linked to the disease. Some scholars have come up with a novel methodology based on implanting endometrial tissues or cells from patients with endometriosis into female nude mice^10,11^ to imitate the pathogenesis and progression of homo endometriosis. These tissues or cells were always separated from ectopic lesions in patients with endometriosis. However, based on the “*Determinant of Uterineeutopic Endometrium*”^12^, the inherent property of eutopic endometrium with endometriosis plays a decisive role, with other factors such as hormone, immune, and local microenvironment acting merely as additional conditions. Furthermore, a growing number of contemporary studies^13–16^ highlight the importance of eutopic endometrium in the pathogenesis of endometriosis.

Menstrual blood-derived stromal cells (MenSCs) are part of the group of endometrial progenitor cells and ideal candidates for reflecting the conditions of the eutopic endometrium. In addition, endometrial stromal cells are proven to participate in the occurrence and development of endometriosis because they enter the pelvic cavity from retrograde menstruation, are often overactivated, and then form new glands and stromal through clonal expansion^17^. Moreover, MenSCs have abundant sources (menstrual blood), and excellent proliferation and auto-transplantation capabilities^18,19^. However, successful endometriotic model approaches using MenSCs have not been reported yet, so whether the discrepancy of MenSCs with and without endometriosis influences the disease occurrence and progress is also a question that remains unanswered.

Therefore, our methodology contrasts the proliferation and migration ability between MenSCs of endometriosis patients (E-MenSCs) and healthy women (H-MenSCs). Further, we explored three kinds of endometriotic models and paired controls related to human eutopic endometrium by implanting E-MenSCs and H-MenSCs. This arrangement might provide a new, unexplored in vivo model to shed light on the endometriotic pathological mechanisms and other insights in pharmacodynamic research.

## Results

### E-MenSCs and H-MenSCs conformed to mesenchymal stromal cell properties

**Figure 1:**
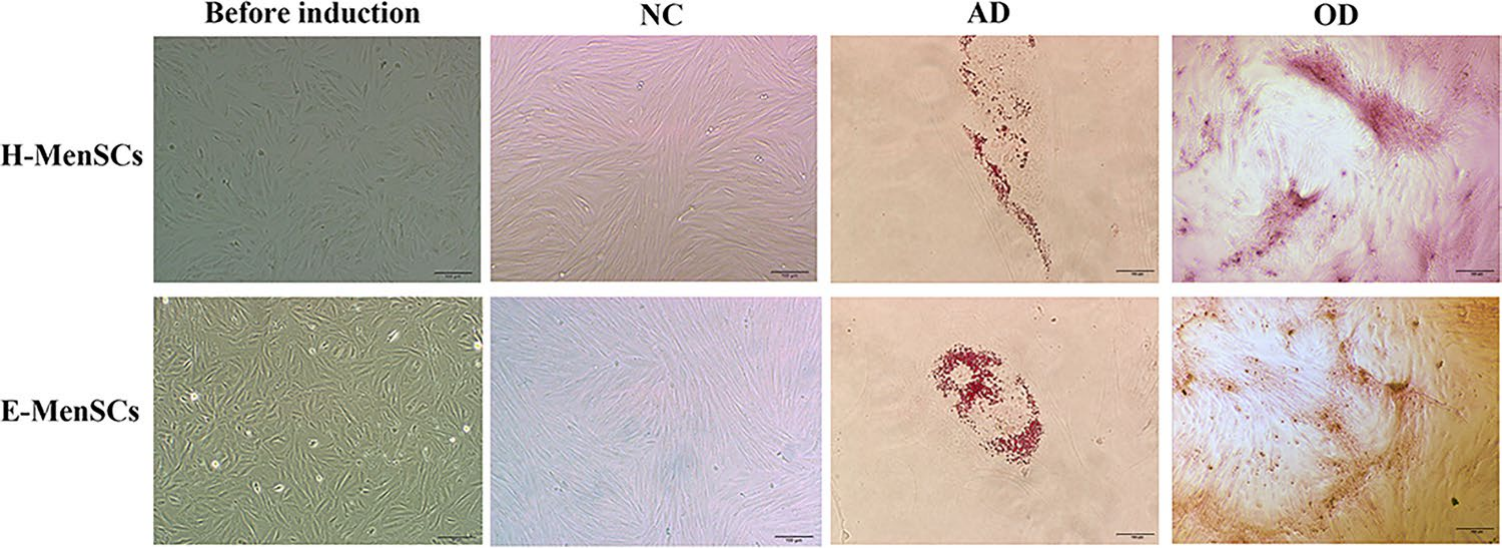
Adipogenic and osteogenic differentiation of MenSCs. Adipogenic and osteogenic differentiation was used for identifying H-MenSCs and E-MenSCs. These cells both showed flat and fibroblast-like morphology and grew dispersedly. Images taken before induction illustrate the original state of H-MenSCs and E-MenSCs (2nd to 4th passage). In the figures, NC (negative control) indicates the development of H-MenSCs and E-MenSCs after 21 days without induction, AD (adipogenic differentiation) depicts MenSCs after adipogenic differentiation, and OD shows the cells after osteogenic differentiation (Scale bar: 100 μm).

E-MenSCs and H-MenSCs exhibited a typical state of endometrial stromal cells (Fig. 1)—a spindle-shaped, fibroblast-like morphology with a radial growth pattern. To identify the mesenchymal stromal cells’ properties of E-MenSCs and H-MenSCs, we detected cell morphology after using adipogenic and osteogenic differentiation. We observed lipid droplets and calcium nodules both in E-MenSCs and H-MenSCs after adipogenic and osteogenic differentiation. In contrast, MenSCs without differentiation maintained the same pattern except for a longer morphology (Fig. 1).

### The same density of E-MenSCs with increased cell proliferation compared to H-MenSCs during 72–120 h

**Figure 2:**
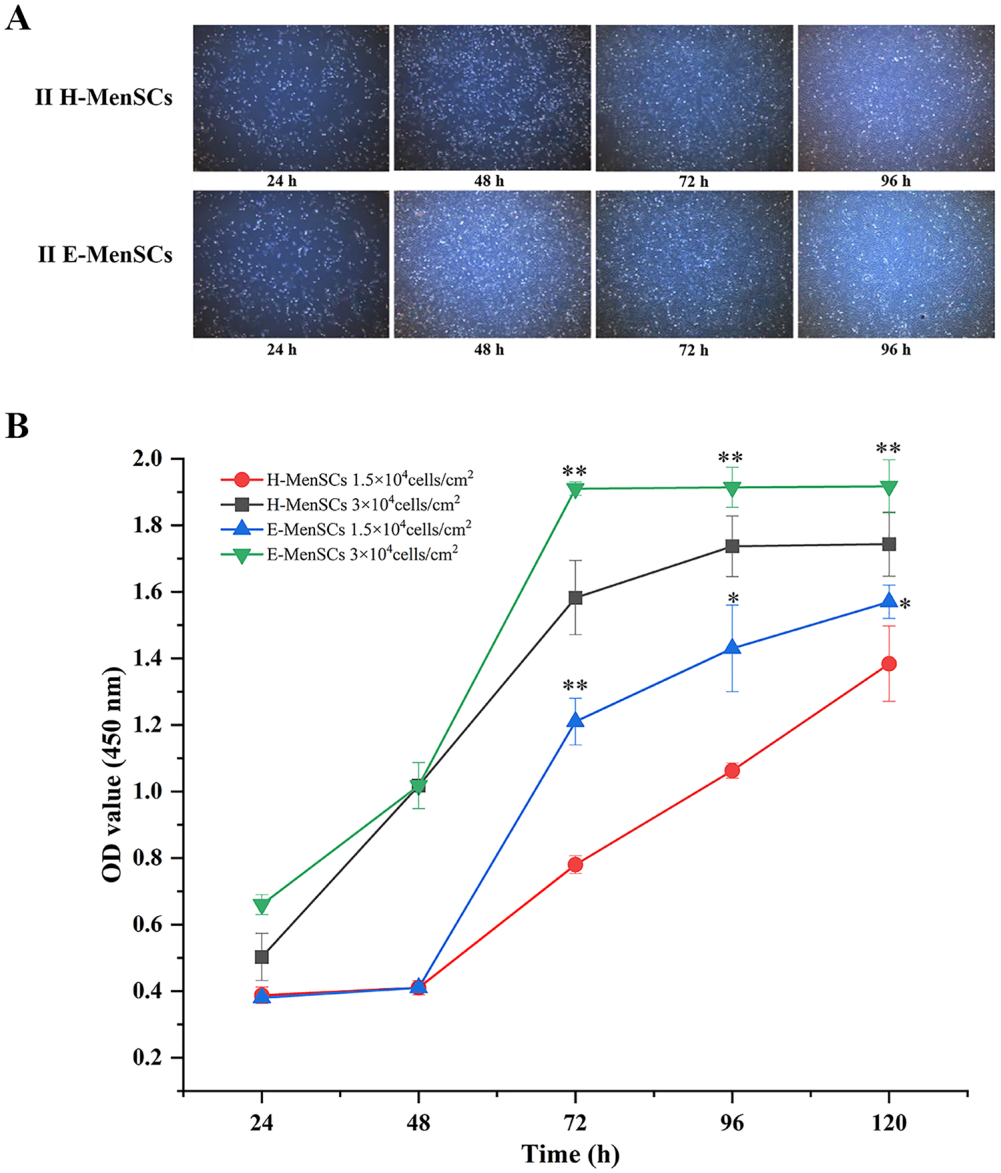
Comparison between E-MenSCs and H-MenSCs on cell proliferation. (**A**) The state of the 2nd passage of H-MenSCs and E-MenSCs from 0 to 96 h. II H-MenSCs: the 2nd passage of healthy menstrual blood-derived stromal cells; II E-MenSCs: the 2nd passage of menstrual blood-derived stromal cells with endometriosis. (**B**) The proliferation of H-MenSCs and E-MenSCs at different time points and cell densities. *P < 0.05, **P < 0.01 vs. H-MenSC group at the same cell density and time point using Student’s *t*-test (*n* = 5). Notes: H-MenSCs: healthy menstrual blood-derived stromal cells; E-MenSCs: menstrual blood-derived stromal cells with endometriosis.

The 2nd passage of E-MenSCs and H-MenSCs generally adhered after 24 h and rapidly proliferated later. E-MenSCs grew faster with 100% confluence at 72 h vs. only 85–90% confluence at 96 h in H-MenSCs (Fig. 2A). As shown in Fig. 2B, E-MenSCs performed enhanced cell proliferation compared to H-MenSCs at 72, 96, and 120 h regardless of the different cell densities—1.5 × 10^4^ cells/cm^2^ (*P* < 0.01, *P* < 0.05, and *P* < 0.05, respectively) or 3 × 10^4^ cells/cm^2^ (*P* < 0.01, *P* < 0.01, and *P* < 0.01, respectively)**.**

### E-MenSCs with enhanced migration and wound-healing capability compared to H-MenSCs

**Figure 3:**
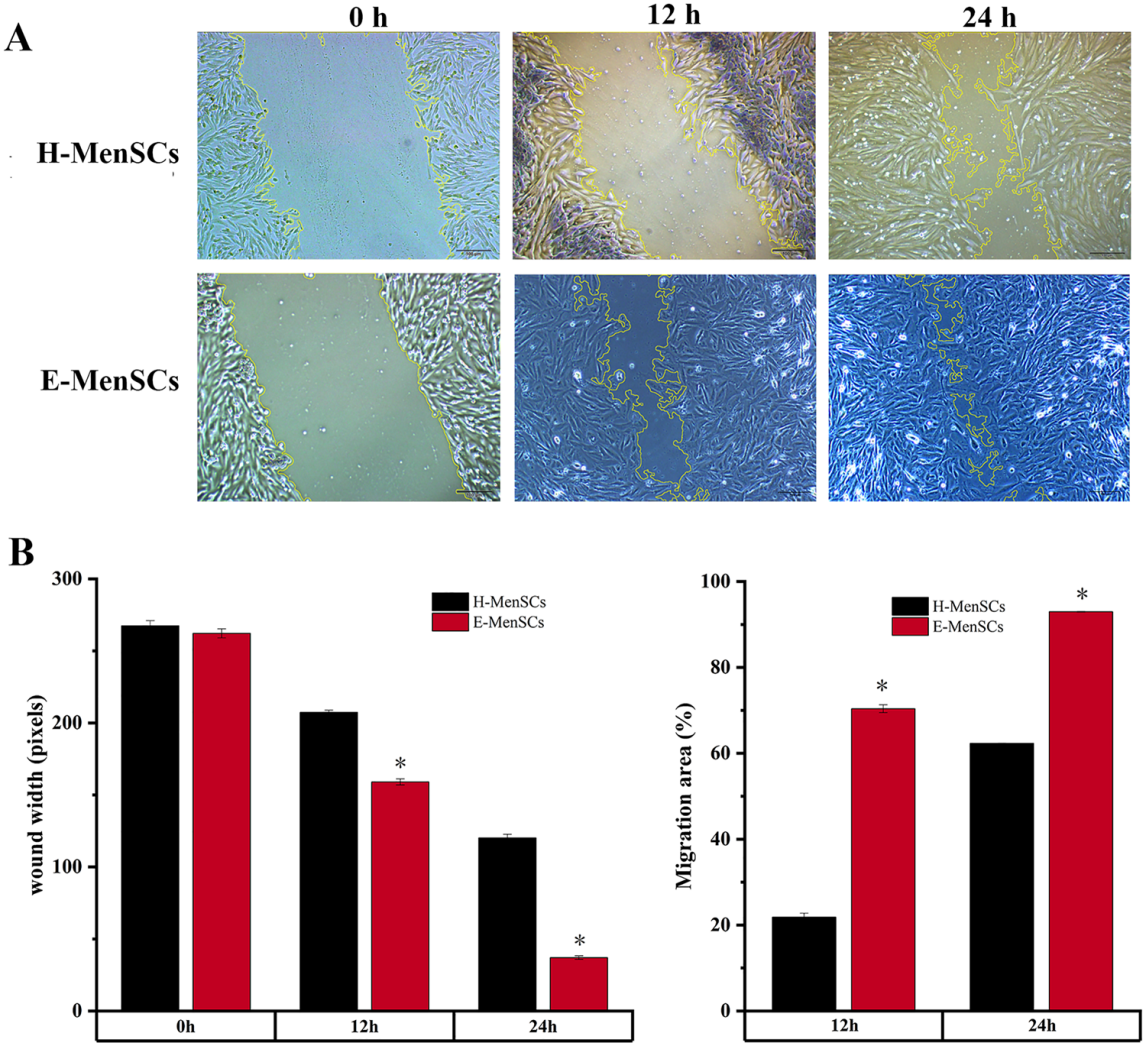
Comparison between E-MenSCs and H-MenSCs on cell migration and wound-healing capability. (**A**) Migration states of H-MenSCs and E-MenSCs. The same scratched area of H-MenSCs and E-MenSCs at 0 h, 24 h, and 48 h was captured by light microscopy. The migration area was highlighted using yellow curves and a scale bar of 100 μm. (**B**) Analysis of wound width and migration area. The wound width (pixels) and migration area (%) were analyzed via ImageJ software. * *P* < 0.05 vs. H-MenSCs at the same time using Student’s *t*-test (*n* = 5). *H-MenSCs* healthy menstrual blood-derived stromal cells, *E-MenSCs* menstrual blood-derived stromal cells with endometriosis.

As illustrated in Fig. 3, the wound width between E-MenSCs and H-MenSCs showed no significant difference at 0 h, but it narrowed down in E-MenSCs at 24 h and 48 h (*P* < 0.05; *P* < 0.05). The presence of more expansive migration areas (after 24 h and 48 h) of E-MenSCs (*P* < 0.05; *P* < 0.05) also indicates enhanced migration and wound-healing capability.

### Lesions observed in E-MenSCs implanting groups—SEM, SCEA, and SCEB

**Figure 4:**
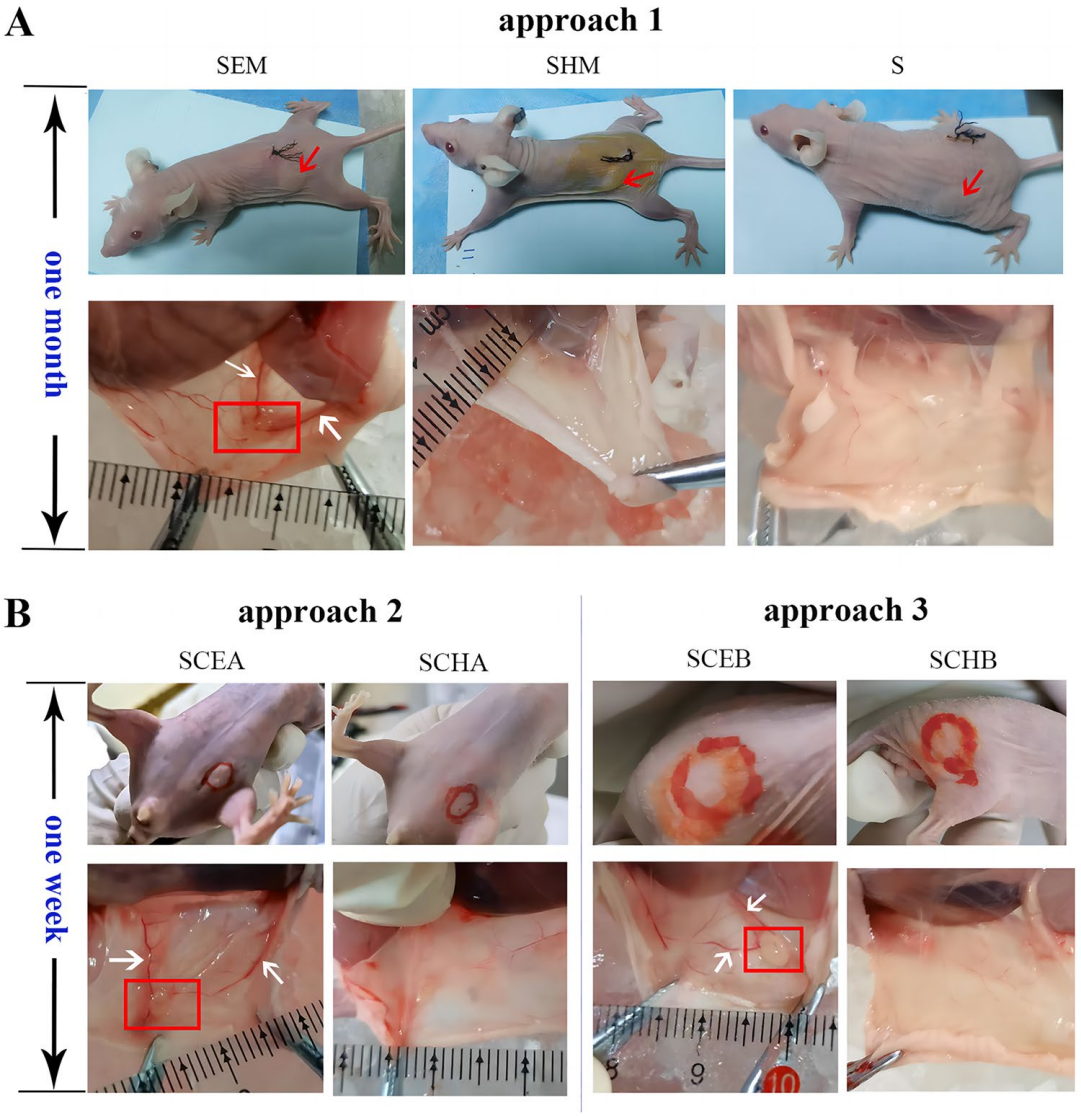
Implants at the beginning and finishing time point after the implantation in seven groups of mice. (**A**) implants in the mice using approach 1 (surgical implantation using scaffolds seeded with MenSCs). The experiments involved three groups: SEM, SHM, and S. The red arrow shows the bulge of implant directly after the implanting operation. The red rectangle highlights the lesion 1 month after the implantation, and the two white arrows mark the surrounded blood vessels. (**B**) Implants in the mice using approaches 2 and 3 (subcutaneous injection of MenSCs in the abdomen and back). Groups were divided into SCEA, SCHA, SCEB, and SCHB. The red circle shows the protrusion hours after the injection. The red rectangle highlights the lesion 1 week after the injection, and the two white arrows mark the surrounded blood vessels. *SEM* scaffolds seeded with E-MenSCs, *SHM* scaffolds seeded with H-MenSCs, *S* scaffolds, *SCEA* subcutaneous injection of E-MenSCs in the abdomen, *SCHA* subcutaneous injection of H-MenSCs in the abdomen, *SCEB* subcutaneous injection of H-MenSCs in the back, *SCHB* subcutaneous injection of H-MenSCs in the back, *E-MenSCs* menstrual blood-derived stromal cells with endometriosis, *H-MenSCs* healthy menstrual blood-derived stromal cells.

**Figure 5:**
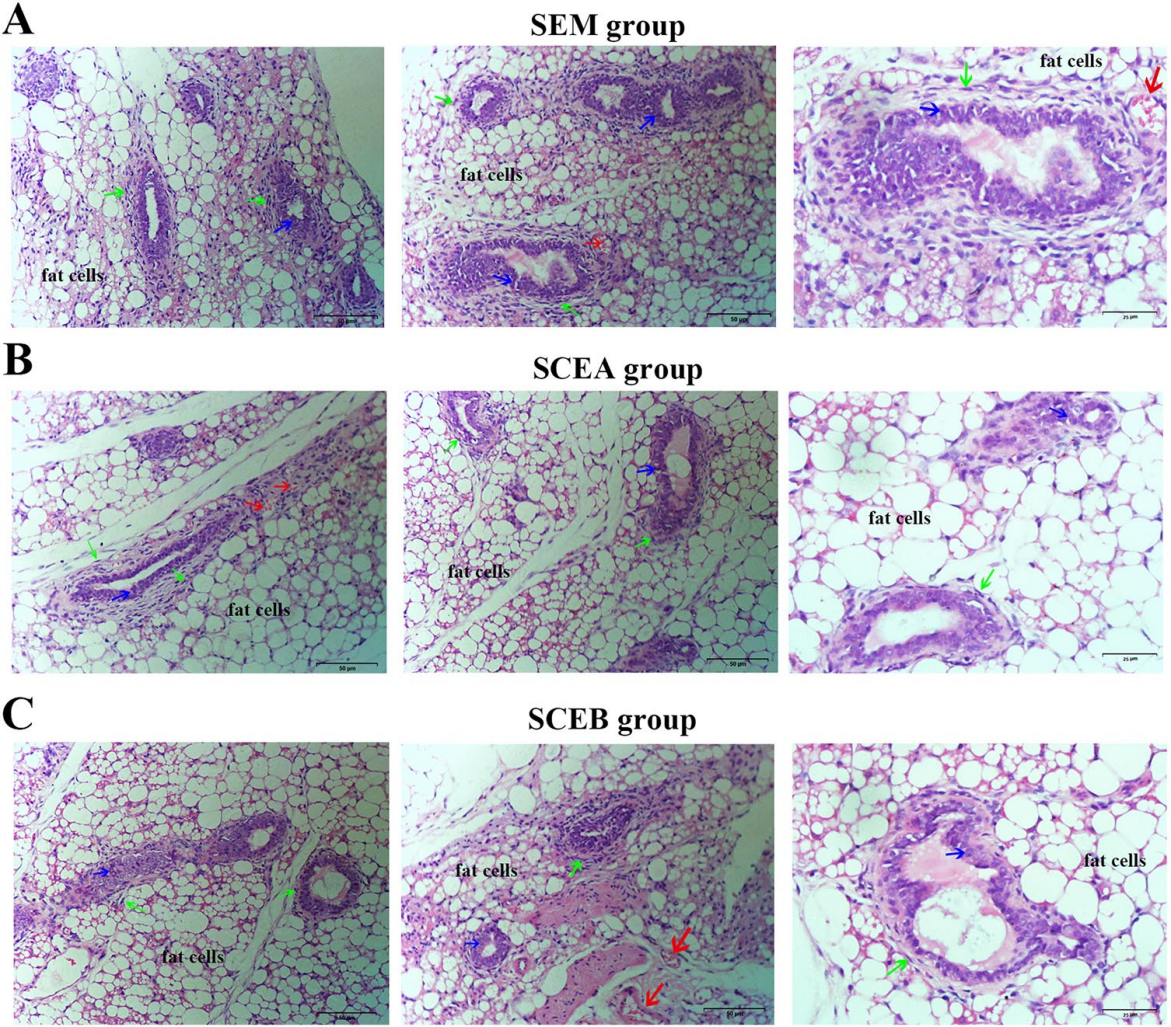
Pathological structure of lesions in E-MenSCs implanting groups: SEM (**A**), SCEA (**B**), and SCEB (**C**) groups. Paraffin-embedded sections of lesions in nude mice of the three groups after hematoxylin and eosin (H&E) staining (Scale bar: 50 μm, 25 μm). Green arrows are pointing to the stromal cells, blue arrows are pointing to glandular epithelial cells (columnar and sponge-like), and red arrows show blood vessels. *SEM* scaffolds seeded with E-MenSCs, *SCEA* subcutaneous injection of E-MenSCs in the abdomen, *SCEB* subcutaneous injection of E-MenSCs in the back.

We observed transparent and cystic spheres (red rectangles) around new blood vessels (white arrows) in the SEM, SCEA, and SCEB groups (Fig. 4), but nothing new was identified in the implanting sites of SHM, S, SCHA, and SCHB groups (Fig. 4). An evident bulge (red arrow) was identified directly after the operation using the approach 1 (surgical implantation using scaffolds seeded with MenSCs), but the implants of S and SHM groups (scaffolds and scaffolds seeded with H-MenSCs) were absorbed completely without blood vessels 1 month after the operation (Fig. 4A). In addition, we found a mung bean-sized protrusion directly after subcutaneous injection in the abdomen (approach 2) and back (approach 3). However, the protrusion (red circle in Fig. 5B) became invisible hours after the injection. SCHA and SCHB groups using H-MenSCs suspension had no tissue formation even 1 week after the injection (Fig. 4B).

We then compared the lesion formation rate of these three approaches. As shown in Table 1, the SCEA group using approach 2 performed the highest (115%). 23 lesions were identified in a total of 20 implantations. The SEM group using approach 1 had a 90% of lesion formation rate, and the SCEB group using approach 3 showed an 80% of lesion formation rate. As for the volume of lesions (Table 2), we found that the lesions in the SEM group, which was approximately equivalent to the attached scaffolds (about 11.1 × 3.2 × 3.5 mm^3^), were the largest among these three groups (*P* < 0.05). In addition, no significant differences were found in the volume of lesions in the SCEA (about 4.2 × 2.8 × 2.4 mm^3^) and SCEB groups (about 4.6 × 2.7 × 2.3 mm^3^) (*P* > 0.05).

**Table 1:** Lesions formation rate in E-MenSCs implanting groups.

Groups	Implant counts	Formation periods (d)	Lesions counts	Lesion formation rate (%)
SEM (*n* = 10)	10	30	9	90
SCEA (*n* = 10)	20	7	23	115
SCEB (*n* = 10)	10	7	8	80

**Table 2:** Lesions’ volumes in E-MenSCs implanting groups (mm^3^, ${\overline{\text{x}}}$ ± s).

Groups	Mice amounts	Lesions counts	Length (mm)	Width (mm)	Height (mm)	Volume (mm^3^)
SEM	10	9	11.11 ± 1.90	3.22 ± 0.67	3.47 ± 0.44	123.60 ± 36.87^#ϕ^
SCEA	10	23	4.22 ± 1.04	2.76 ± 0.63	2.44 ± 0.58	27.37 ± 9.10*
SCEB	10	8	4.63 ± 1.19	2.69 ± 0.80	2.26 ± 0.32	29.56 ± 14.05*

Data were expressed as mean ± standard deviation using one-way ANOVA (*n* = 10). *P < 0.05 vs*.* the SEM group; ^#^*P* < 0.05 vs. the SCEA group; ^ϕ^*P* < 0.05 vs. the SCEB group. *SEM* scaffolds seeded with E-MenSCs, *SCEA* subcutaneous injection of E-MenSCs in the abdomen, *SCEB* subcutaneous injection of E-MenSCs in the back.

### Endometrial glands and stroma observed in the lesions

In the SEM, SCEA, and SCEB groups, we observed endometrial glands and stroma connected with fatty tissue in their lesions’ tissue section using HE staining (Fig. 5). Glands consisted of many columnar glandular epithelial cells (blue arrow) and were tightly surrounded by stromal cells (green arrow). We also identified secretions in the glands and subcutaneous fat cells forming the fibrous fatty connective tissue, as illustrated in Fig. 5. In addition, some red blood cells (red arrow) were also observed in these tissue sections. Thus, we conclude that most of the glands in endometriosis were organized orderly and consisted of the acini. In addition, these three approaches of endometriotic models also provide evidence that MenSCs could induce angiogenesis and form ectopic lesions.

### Lesions originated from humans, not mice

**Figure 6:**
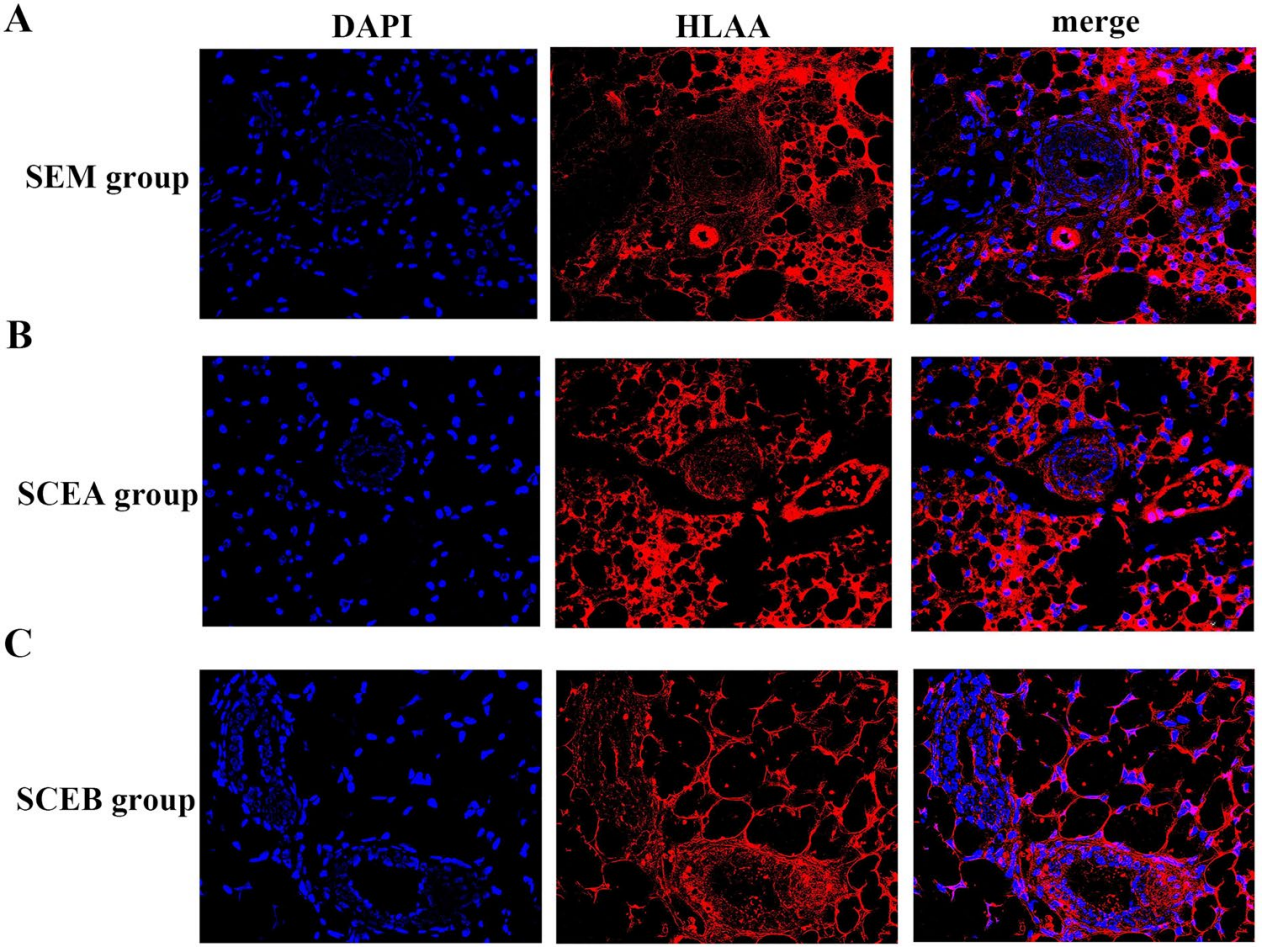
*HLAA* immunofluorescent staining of ectopic lesions from SEM (**A**), SCEA (**B**), and SCEB (**C**) groups. DAPI (blue fluorescent) represents the nucleus in the tissues. The images confirm that HLAA (red fluorescent) was expressed in the lesions of all three groups. The last pictures in the row is a merging process of both in a scale bar of 100 μm. *SEM* scaffolds seeded with E-MenSCs, *SCEA* subcutaneous injection of E-MenSCs in the abdomen, *SCEB* subcutaneous injection of E-MenSCs in the back, *DAPI* diamidino-phenyl-indole, *HLAA* human leukocyte antigen α.

To confirm that the lesions in female SCID mice originated from implanted human cells instead of mice, we analyzed *HLAA*—uniquely expressed on human cell membranes but not on mouse cells—expression in those lesions using immunofluorescent. Unambiguously, the endometriotic ectopic lesions in these three groups using three approaches were all identified by positive human *HLAA* in SCID mice (Fig. 6). The results thus confirm that those lesions originated from human E-MenSCs.

## Discussion

The most important outcomes of our experiments confirmed that E-MenSCs performed increased proliferation, migration, and wound-healing capability compared to H-MenSCs. E-MenSC also formed experimental lesions in the nude mice using three different approaches, while H-MenSCs did not. Thus, our study demonstrates the successful preparation of endometriotic nude mice models by implanting E-MenSCs using three different approaches. Moreover, we demonstrated that H-MenSCs implants had no visible influence on nude mice—no new tissues were observed at the implanting area 1 week or 1 month after the implantation. Liu’s research^19^ reported a similar phenomenon: nude mice that received H-MenSCs remained tumor-free until week 12 after the experiment. Furthermore, no significant alteration was observed in the liver, heart, and kidney during the H-MenSCs transfer. We inferred the reasons why H-MenSCs groups formed nothing in the implanting area were as follows: (i) nude mice were immunodeficient strains with host tolerance; (ii) H-MenSCs impose little immunogenicity^20,21^ and tumorigenicity after the transfer^22^; (iii) high apoptosis potential of H-MenSCs^23^; (iv) the relatively few stromal cell densities in H-MenSCs.

The eutopic endometrium is the primary factor in the development of endometriosis^13,14,24,25^. When MenSCs were isolated from the shed inner uterine lining (menstrual blood), they can be used as the potential in-vitro model to reflect the characteristics of a eutopic endometrium^26^. The use of MenSCs in an in-vitro model has some important advantages such as abundant and periodic origin, mature isolation technology (without surgical intervention), and apparent mesenchymal stromal cell properties. In the present study, we isolated and compared E-MenSCs and H-MenSCs from patients with endometriosis and healthy volunteers and this is a novel approach, compared to the methodologies used by recent studies focused on the same phenomenon. In our model, we found that the excellent proliferation and migration ability of E-MenSCs might promote the attachment, aggression, and angiogenesis (AAA) of endometrial stromal cells and lead to endometriotic ectopic lesions. Nikoo et al.^27^ compared E-MenSCs and MenSCs from none-endometriosis undergoing laparoscopy for tubal ligation (NE-MenSCs), and they also found E-MenSCs had higher proliferation and invasion potentials compared to NE-MenSCs using ^3^H-thymidine incorporation and Matrigel invasion assay. The present study compared E-MenSCs with MenSCs from healthy volunteers focused on the dynamic proliferation during 24–120 h time points and migration capability at 0 h, 12 h, and 24 h time points using CCK-8 assay and wound-healing assay. In addition, the present study further implanted them into nude mice and observed the formation rate of ectopic lesions at the implanting area to compare their invasion potential. No new tissue formed among the H-MenSCs implanting groups and over 80% lesion formation rate among the E-MenSCs implanting groups also indicated the excellent invasion potentials of E-MenSCs.

In addition, a new contemporary study^28^ indicates that E-MenSCs had higher expression of *Cyclin D1* (a cell cycle-related gene), *MMP2* and *MMP9* (migration- and invasion-related genes), and *SOX2* and *SALL4* (stemness genes) genes compared with H-MenSCs. In our research, we demonstrated that implanting cell density truly played a role in cell proliferation ability: the proliferation ability of H-MenSCs (1 × 10^5^ cells/mL) surpassed E-MenSCs with lower density (5 × 10^4^ cells/mL), while E-MenSCs are still superior to H-MenSCs at the same lower density (5 × 10^4^ cells/mL).

To exclude the influence of the self-renewal ability of MenSCs as well as their potential to maintain stem cells properties from donor's age and passage number^29^, 2nd to 4th passage cells were selected for the experiments, and the donors were analogous without significant difference (age 26–36 years; BMI 18–28 kg/m^2^).

**Figure 7:**
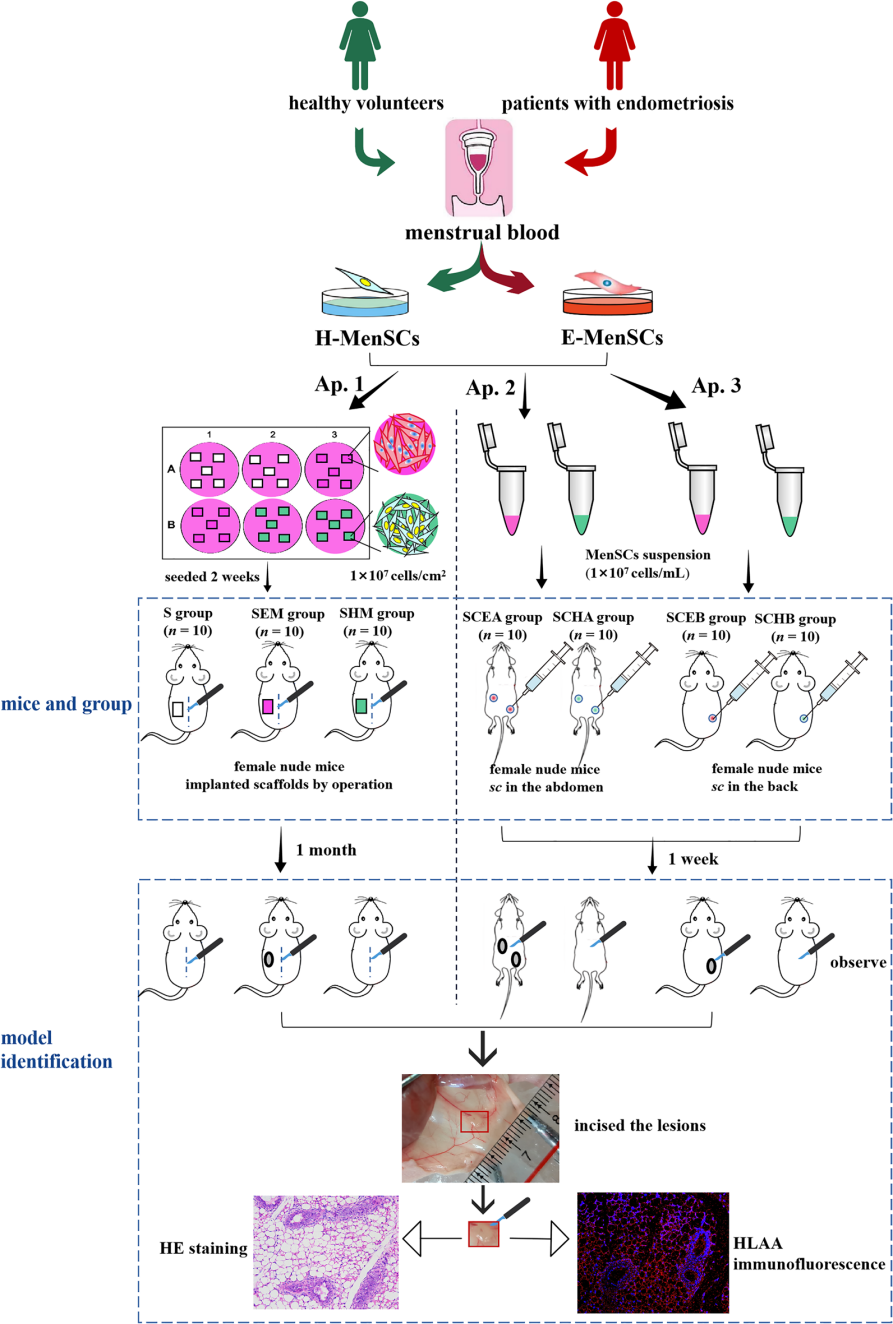
Graphic protocols for the three approaches of in vivo endometriotic model by MenSCs implantation. *H-MenSCs* menstrual blood-derived stromal cells from healthy volunteers, *E-MenSCs* menstrual blood-derived stromal cells with endometriosis, *Ap. 1* approach 1—surgical implantation using scaffolds seeded with MenSCs, *Ap. 2* approach 2—subcutaneous injection of MenSCs in the abdomen, *Ap. 3* approach 3—subcutaneous injection of MenSCs in the back, *S* scaffolds, *SEM* scaffolds seeded with E-MenSCs, *SHM* scaffolds seeded with H-MenSCs, *SCEA* subcutaneous injection of E-MenSCs in the abdomen, *SCHA* subcutaneous injection of H-MenSCs in the abdomen, *SCEB* subcutaneous injection of E-MenSCs in the back, *SCHB* subcutaneous injection of H-MenSCs in the back, *sc* subcutaneous injection, *HE staining* hematoxylin–eosin staining, *HLAA* human leukocyte antigen α.

Given the excellent proliferation and migration ability of E-MenSCs, we then implanted E-MenSCs using three approaches into nude mice and prepared novel endometriotic in vivo models (Fig. 7). These models were all successful and presented noticeable advantages and disadvantages. The primary advantages of subcutaneous injection (approaches 2 and 3) are that the procedure is non-invasive, safe, and simple, with a short modeling period (1 week), and a lesions formation rate of over 80%. Moreover, the lesions formation rate using approach 2 (subcutaneous injection in the abdomen) is 115%: 23 lesions were finally formed 1 week after 20 injections. The phenomenon might have benefited from the sufficient blood supply and fat deposits of the abdomen, which is essential to the formation and metastasis of ectopic lesions^30,31^. Abundant capillaries and lymphatic vessels connecting blood, lymph, and nerves of abdominal organs in the peritoneum support the nutrition and energy for lesion growth. The abdomen is also the dominant implanting position of tumor-bearing nude mice^32–35^. However, it is still challenging to observe the implants outside the nude mice because the implant (mungbean protuberance) tends to be absorbed within hours. We only euthanized the nude mice and expose the implantation area to identify the lesions formation. Despite the shortcomings, our procedures could still successfully verify the high lesion formation rate. In addition, this endometriotic model also can be used for evaluating drugs against endometriosis and exploring their mechanisms for the disease. we have explored the effect and mechanism of a Chinese classical remedy (Hupo Powder) against endometriosis in vivo by the endometriotic model using approach 2^36^.

As for approach 1 (surgical implantation using scaffolds seeded with MenSCs), the main advantages are the stable lesion volumes (largely consistent with the scaffold) and the obvious bulge of implants (visible outside the nude mice). However, it is important to consider that the method has serious disadvantages, such as the long modeling period (2 months), and complicated and invasive procedures, especially in immune-deficient nude mice. But the safe also can be assured with the skilled operations that during these experiments no mice died.

As for the potential application of these endometriotic models, we suggest that these models can be used for exploring the underlying role of eutopic endometrium in the pathogenesis, progress, and intervention of endometriosis. For instance, regarding the phenomenon of “progesterone resistance” in patients with endometriosis^37^, including 1/4–1/3 patients with endometriosis are no reaction to the first-line drug—combined oral contraceptives^38^, more and more studies indicate that eutopic endometrium in endometriosis is required to be further explored^21,39,40^. Some progesterone receptors (progesterone and adipoQ receptor 8 (*PAQR8*) and progesterone and adipoQ receptor 6 (*PAQR6*)) are only reduced in eutopic endometrium and no significant difference between ectopic endometrium and the endometrium of women without endometriosis^41^. The progesterone receptors (PRs) change in eutopic endometrium might be the cause, while the PRs change in ectopic endometrium is the phenomenon^24^. Thus, this type of endometriotic model and paired control model could be used to unearth the cause of progesterone inaction in endometriosis and promote the development of novel therapeutics for the prevention and intervention of the disease.

The present study has some limitations worth mentioning. First, we identified MenSCs’ stromal cell properties by morphology observation, adipogenesis, and osteogenesis differentiation. The surface markers (CD105, CD73, CD90. CD45, CD34, CD14, CD19) of mesenchymal stem cells still need to be further detected in E-MenSCs and H-MenSCs to further validate the results. In addition, we used H&E staining and *HLAA* immunofluorescence to evaluate the modeling process, so the lesions’ pathological structures followed the properties of the endometrial tissue and originated from humans, not mice. In the future, more markers are required to identify ectopic endometriotic lesions in the explanted tissue, including a human glandular epithelial marker (EpCAM, cytokeratin) and a human stromal marker (CD10, CD140b, vimentin). As for H-MenSCs and scaffolds implanted in nude mice, we only observed the implanting area and found no significant differences. To explore the inherent change, we will label the sites just after the implantation, slice the implanting area 1 week or 1 month after the procedure, and detect human indexes and inflammatory cytokines to ensure these nude mice were indeed free of lesions and inflammations in the local implanting sites. The nude mice that received H-MenSCs or scaffolds could be suitable negative controls in the future.

Therefore, this article focused on comparing the properties of H-MenSCs and E-MenSCs on proliferation and migration/invasion ability in vitro and in vivo. Based on the outcomes, we originated three novel implanting endometriotic models to reflect the characteristics of the eutopic endometrium with endometriosis. These novel models provide a new foundation for interpreting endometriotic pathology and discussing suitable interventions for diagnosed patients.

## Materials and methods

### Menstrual blood collection of patients with endometriosis and healthy volunteers

This experimental study was approved by the Institutional Review Board of Beijing University of Chinese Medicine Third Affiliated Hospital (BZYSY-2021KYKTPJ-12). In addition, all experiments were performed in accordance with relevant guidelines and regulations. The patients with endometriosis were diagnosed by the classical clinical symptoms (progressive dysmenorrhea, chronic pelvic pain, or coital pain), and overexpression of serum CA-125, infertility, or findings of endometriomas by transvaginal ultrasound (TVU). Patients were all referred to the gynecology department of Beijing University of Chinese Medicine Third Affiliated Hospital from Dec. 2020 to Apr. 2022. Healthy volunteers were all graduate students from the Beijing University of Chinese Medicine. All participants signed the Informed Consent. We checked graduate students’ Freshmen Enrolment Physical Examinations and detected their pelvic cavities by TVU to identify their healthy bodies. We considered healthy volunteers as the control group (*n* = 6), and patients with endometriosis were allocated to the model group (*n* = 6). We enrolled participants according to the inclusion criteria: (i) age range between 26 and 36 years, (ii) body mass index (BMI) of 18–28 kg/m^2^, (iii) no hormonal treatments for at least 6 months, (iv) no anti-endometriosis drugs for at least 1 month, (v) no endometriosis-associated surgery before the experiment, and (vi) no history of malignant tumor or autoimmune diseases. As shown in Fig. 1, each group's menstrual blood (≥ 5 mL) was collected on the 2nd day of the cycle using menstrual cups. Then, the blood samples were rapidly transferred into sterile 50-mL centrifuge tubes under laboratory conditions after ice transportation and MenSCs were isolated within 2 h.

### Isolation and culture of E-MenSCs and H-MenSCs

After mixing with the equivalent volume of phosphate-buffered saline (PBS; Aoqing Biotechnology, Beijing, China, AQ10010) and slowly adding lymphocyte stratified liquid (Haoyang Biological Manufacture, Tianjin, China, LTS1077), we then separated mononuclear cells of menstrual blood by density gradient centrifugation at 2000 r/min for 10 min. Cells from the middle layer were extracted to a new tube and washed twice with PBS before we removed the supernatant. Subsequently, the cells were resuspended and cultured in DMEM/F12 medium (1:1; Aoqing Biotechnology, Beijing, China, AQ11330) containing 10% fetal bovine serum (FBS; Aoqing Biotechnology, Beijing, China, AQmv09900) and 1% penicillin/streptomycin (Aoqing Biotechnology, Beijing, China, AQ512) that were mixed evenly, counted and adjusted to an appropriate density (3 × 10^4^ cells/cm^2^) for cell culture.

We cultured the selected cells for 72 h at 37 °C in a humidified 5% CO_2_ atmosphere, and the culture medium was changed every 3 days. When cell confluence reached 80–90%, the cells were passaged using 0.25% Trypsin–EDTA (Aoqing Biotechnology, Beijing, China, AQ515). With the suspended cells being removed by trypsin digestion for liquid and passage, MenSCs were isolated and purified for about 7–14 days, using the law adherent method. To avoid the difference from the cell passage and maintain the characteristics of mesenchymal stromal cells, we selected the 2nd to 4th passage cells for subsequent experiments. We could isolate 2–8 cell culture bottles (75 mL) of MenSCs after 2–4 passages from 5 mL menstrual blood with a cell density of 1 × 10^7^ cells/cm^2^ per bottle. The menstrual blood of six patients with endometriosis and six healthy volunteers was adequate to form this study’s experiments.

### Mesenchymal stromal cell identification of E-MenSCs and H-MenSCs

Adipogenic and osteogenic differentiation were used for identifying the mesenchymal stromal cells’ properties of E-MenSCs and H-MenSCs. For adipogenic differentiation, the 2nd to 4th passages of E-MenSCs and H-MenSCs were seeded into a 6-well plate at a density of 3 × 10^4^ cells/cm^2^ and cultured at 37 °C in a humidified 5% CO_2_ atmosphere. Sub-confluent cells were induced using adipogenic medium (iCell Bioscience, Shanghai, China, iCell-MSCYD-004) containing DMEM/F12 medium, 10% FBS, 1% penicillin–streptomycin, 1% glutamine, 0.2% insulin, 0.1% rosiglitazone, 0.1% dexamethasone, and 0.1% 3-isobutyl-1-methylxanthine. The induction continued for 21 days with renewed medium every 3 days. After that, the differentiated we exposed the E-MenSCs and H-MenSCs by staining the fatty vacuoles with Oil Red O (Beyotime Biotechnology, Shanghai, China, C0158S).

For osteogenic differentiation, E-MenSCs and H-MenSCs were also seeded into a 6-well plate at a density of 3 × 10^4^ cells/cm^2^. Osteogenic differentiation medium (iCell Bioscience, Shanghai, China, iCell-MSCYD-002) was added after the cells reached sub-confluence, consisting of DMEM/F12 medium, 10% FBS, 1% penicillin–streptomycin, 1% glutamine, 1% β-Sodium glycerophosphate, 0.2% ascorbic acid, and 0.01% dexamethasone for 21 days. Then, we evaluated the osteogenesis by light microscopy (Olympus, Tokyo, Japan, CKX53) after staining the cells with Alizarin Red S (Beyotime Biotechnology, Shanghai, China, C0140).

Undifferentiated cells were used as controls, and the experiments were performed in triplicates.

### Proliferation assay

We conducted a comparative study of cell proliferation between E-MenSCs and H-MenSCs using a cell counting kit-8 (CCK-8) assay. Images of the 2nd passage E-MenSCs and H-MenSCs were collected after being cultured for 24, 48, 72, and 96 h using a microscope. The 2nd–4th passage cells (1.5 × 10^4^ cells/cm^2^ and 3 × 10^4^ cells/cm^2^) were cultured for 24, 48, 72, 96, and 120 h, respectively (*n* = 5). 10 µL CCK-8 solution (Aoqing Biotechnology, Beijing, China, AQ308) was added to each well and cells were then incubated for 4 h at 37 °C. The optical density (OD) of each well was measured using a microplate reader (Thermo Fisher Scientific, Waltham, USA, A51119500C) at 450 nm. Origin 2021 (OriginLab, Northampton, MA) was used to plot proliferation curves.

### Wound healing assay

We also conducted a comparative study of cell migration between E-MenSCs and H-MenSCs using a wound healing assay. First, the cells (3 × 10^5^ cells/cm^2^) were seeded into a 6-well plate (*n* = 5) and when cell confluence reached 90–95%, we scratched a straight line using a 10 μl pipette tip. After that, we discarded the original medium and washed every well with PBS to remove the detached cells. Then, DMEM medium without serum (Invitrogen, Carlsbad, California, USA, 31985062) was added to these wells. We photographed the scratched area 0, 24, and 48 h after the scratches under a phase-contrast microscope. Then, the wound width and migration area were analyzed by ImageJ and plotted to a histogram using Origin 2021.

### Mice and group

This animal study was carried out following the ARRIVE guidelines 2.0. Seventy female nude mice (BALB/cAnN. Cg-Foxn1nu/CrlNarl, 4 weeks of age, 20–22 g of weight) were purchased from Beijing Vital River Laboratory Animal Technology Co., Ltd (license No. SCXK 2021-0006). These mice were housed in the laboratory of Beijing University of Chinese Medicine, the specific pathogen-free laboratory center under standard conditions with a light/dark cycle of 12/12 h and allowed ad libitum access to sterilized feed and water. After 1 week of acclimatization, as shown in Fig. 7, we randomly divided them into seven equal-sized groups (*n* = 10) using a random number table, containing scaffolds seeded with E-MenSCs (SEM) (*n* = 10), scaffolds seeded with H-MenSCs (SHM) (*n* = 10), and just scaffolds (S) (*n* = 10); The different groups underwent subcutaneous injection of E-MenSCs in the abdomen (SCEA) (*n* = 10), subcutaneous injection of H-MenSCs in the abdomen (SCHA) (*n* = 10), subcutaneous injection of E-MenSCs in the back (SCEB) (*n* = 10), and subcutaneous injection of H-MenSCs in the back (SCHB) groups (*n* = 10). H-MenSCs implanting groups (SHM, SCHA, and SCHB groups) and the S group were the controls. Then 1 month of SEM, SHM, and S groups after the implantation and 1 week of SCEA, SCHA, SCEB, and SCHB groups after the implantation, these mice were humanely killed by cervical dislocation, followed by the implants observation, ectopic lesions collection, and modeling evaluation using hematoxylin–eosin (H&E) staining and immunofluorescent staining.

All animal experiments were approved by the Ethics Committee of Beijing University of Chinese Medicine (No. BUCM-4-2021042003-2131) and were performed under relevant guidelines and regulations.

### Three approaches of in vivo endometriotic model by MenSCs implantation

As mentioned above, E-MenSCs and H-MenSCs were isolated from the menstrual blood of six patients with endometriosis and six healthy volunteers, respectively. We used the 2nd to 4th passage of cells for this experiment and cell suspensions (1 × 10^7^ cells/cm^2^) were prepared using the DMEM/F12 medium (including 20% FBS and 1% penicillin–streptomycin). Figure 7 shows the protocols of these three approaches in detail.

### Approach 1 (Ap. 1): surgical implantation using scaffolds seeded with MenSCs

We used the gelatin sponges (Jiangxi Zhongqiang Industrial Co., Ltd., China) as the scaffold and divided one of them (60 mm × 20 mm × 5 mm) into six pieces (20 mm × 20 mm × 2.5 mm per scaffold). All of them were soaked overnight in DMEM/F12 medium containing 20% FBS and 1% penicillin–streptomycin. Then, 50 μL E-MenSCs and H-MenSCs suspensions (1 × 10^7^ cells/cm^2^) were dropped on each scaffold with pipettes and cultured for 2 weeks for adequate adherence at 37 °C in a humidified 5% CO_2_ atmosphere^42^. A scaffold seeded with cells was surgically implanted in the left back of female nude mice. S group mice (*n* = 10) were implanted with a scaffold without cells, SEM group mice (*n* = 10) were implanted with a scaffold seeded with E-MenSCs, and SHM group mice (*n* = 10) were implanted with a scaffold seeded with H-MenSCs.

The operation procedures were as follows: (i) the mice were anesthetized with 1% sodium pentobarbital (50 mg/kg) using intraperitoneal injection, and the skin of the back was disinfected; (ii) using small scissors, we made an incision of around 1 cm in the middle of the back skin; (iii) a scaffold was implanted in each animal's subcutaneous layer of the left back under sterile conditions. About 1 month after the operation, the ectopic lesions and angiogenesis could still be observed, but the scaffold was almost completely absorbed^10^.

### Approach 2 (Ap. 2): subcutaneous injection of MenSCs in the abdomen

We subcutaneously injected 100 μL E-MenSCs and H-MenSCs cell suspensions (1 × 10^7^ cells/mL) into the abdomen on both sides of mice with a 1 mL syringe (Fig. 1). SCEA group (*n* = 10) was injected with E-MenSCs, while SCHA group (*n* = 10) was injected with H-MenSCs. According to Su's guidelines^11^ of pre-experiments, the ectopic lesions were observed 1 week after the injection.

### Approach 3 (Ap. 3): subcutaneous injection of MenSCs in the back

We subcutaneously injected 50 μL E-MenSCs and H-MenSCs cell suspensions (1 × 10^7^ cells/mL) into the right back of mice with a 1 mL syringe (Fig. 1). SCEB group (*n* = 10) was injected with E-MenSCs, while SCHB group (*n* = 10) was injected with H-MenSCs. The ectopic lesions were also observed 1 week after the injection.

### Model evaluation

The endometriotic models were evaluated by three aspects: (i) ectopic lesions and peripheral angiogenesis were observed at the implantation area; (ii) endometrial glands and stroma were both observed in the lesions’ histologic examination; (iii) *HLAA* expressed in these ectopic lesions.

### Observation of ectopic lesions formation

Mice using Ap. 1 were killed by cervical dislocation 1 month after implantation, while mice using Ap. 2 and Ap. 3 were killed 1 week after injection. The implantation positions were incised to entirely expose the implants. Then, we observed and examined the lesions and peripheral angiogenesis and collected images. A digital caliper was used to measure the length, width, and height of the ectopic lesions.

### Pathological structure of ectopic lesions using hematoxylin–eosin (H&E) staining

The implants were harvested carefully for histologic examination after H&E staining. The morphology of tissues, cells, and blood vessels could be observed in detail under a light microscope. It is noteworthy that our examination mainly focused on whether we could identify endometrial glands and stroma or not.

### *HLAA* expression in ectopic lesions by immunofluorescent staining

*HLAA* is a specific protein of humans and it is not part of the organism of female nude mice. We used immunofluorescent staining of *HLAA* to identify whether the implants originated from humans^10^. Paraffin-embedded sections of the implants were dewaxed, antigen repaired, blocked, and incubated with *HLAA* antibody (1:100, Abcam, ab52922) in a humidified chamber at 4 °C overnight, then washed with PBS and incubated with the secondary antibody—Goat anti-Rabbit (Alexa Fluor^®^594) (1:200, Abcam, ab150080) for 2 h at 37 °C in the dark. After removing the secondary antibody solution and washing it with PBS, the cell nucleus was stained with 4′,6-diamidino-2-phenylindole (DAPI, Abcam, ab285390), and tissues were sealed with anti-fluorescence-quenching sealants. Fluorescence microscopy (Olympus) was used to observe and collect the official images.

### Statistical analysis

Statistical analysis was carried out using IBM SPSS Statistics 21 software (IBM Corp, Armonk, NY). The experimental data are presented as the mean ± standard deviation (SD). One-way ANOVA analysis was used for the comparison among groups, and the Student’s *t*-test was used between the two groups. The threshold for statistical significance was established at *P* < 0.05.

### Ethics approval and consent to participate

Experiments with human material were approved by the Institutional Review Board of Beijing University of Chinese Medicine Third Affiliated Hospital (BZYSY-2021KYKTPJ-12, Beijing, China). All participants signed informed consent. All animal experiments were approved by the Ethics Committee of Beijing University of Chinese Medicine (BUCM-4-2021042003-2131).

## Data Availability

The datasets used and/or analysed during the current study are available from the corresponding author on reasonable request.

## Abbreviations

MenSCs: Menstrual blood-derived stromal cells
E-MenSCs: Menstrual blood-derived stromal cells with endometriosis
H-MenSCs: Healthy menstrual blood-derived stromal cells
Ap. 1: Approach1—surgical implantation using scaffolds seeded with MenSCs
Ap. 2: Approach2—subcutaneous injection of MenSCs in the abdomen
Ap. 3: Approach3—subcutaneous injection of MenSCs in the back
S: Scaffolds
SEM: Scaffolds seeded with E-MenSCs
SHM: Scaffolds seeded with H-MenSCs
SCEA: Subcutaneous injection of E-MenSCs in the abdomen
SCHA: Subcutaneous injection of H-MenSCs in the abdomen
SCEB: Subcutaneous injection of E-MenSCs in the back
SCHB: Subcutaneous injection of H-MenSCs in the back
sc: Subcutaneous injection
H&E: Hematoxylin–eosin
*HLAA*: Human leukocyte antigen α
