# Author Correction: Novel in vivo endometriotic models associated eutopic endometrium by implanting menstrual blood-derived stromal cells from patients with endometriosis

**DOI:** 10.1038/s41598-024-55394-x

**Published:** 2024-02-28

**Authors:** Yuejian Zhang, Tiantian He, Taoxiu Lin, Qi Guo, Chaoyue Huo, Song Ze Roberts, Mengping Yang, Sichen Yang, Luyi Gao, Wenjuan Zhang, Changxiang Li, Xiaona Ma

**Affiliations:** 1 The Third School of Clinical Medicine, Beijing University of Chinese Medicine, Beijing, China; 2 Department of Galactophore, Beijing University of Chinese Medicine Affiliated Third Hospital, Beijing, China; 3 The School of Traditional Chinese Medicine, Beijing University of Chinese Medicine, No. 11. Beisanhuang Dong Street, Chaoyang District, Beijing, 100029 China; 4 Department of Gynecology, Beijing University of Chinese Medicine Affiliated Third Hospital, No. 51. Xiaoguan Street, Chaoyang District, Beijing, 100029 China

Correction to: *Scientific Reports* 10.1038/s41598-023-35373-4, published online 23 May 2023

The original version of this Article contained errors in Figure 5.

As a result of errors during figure assembly, the second panel of 5A (SEM) group was duplicated from the second panel of 5B (SCEA) group. Additionally, the third panel of 5B (SCEA group) was partially duplicated from the first panel of 5C (SCEB group). The original Figure 5 and accompanying legend appear below.

**Figure 5:**
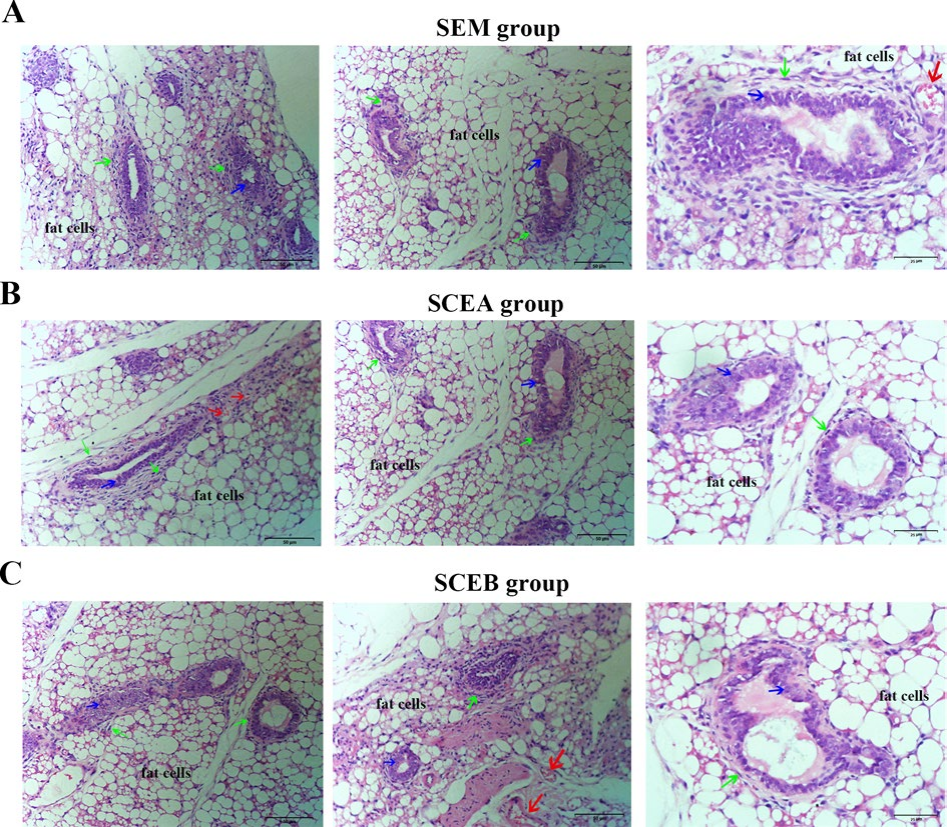
Pathological structure of lesions in E-MenSCs implanting groups: SEM (**A**), SCEA (**B**), and SCEB (**C**) groups. Paraffin-embedded sections of lesions in nude mice of the three groups after hematoxylin and eosin (H&E) staining (Scale bar: 50 μm, 25 μm). Green arrows are pointing to the stromal cells, blue arrows are pointing to glandular epithelial cells (columnar and sponge-like), and red arrows show blood vessels. SEM scaffolds seeded with E-MenSCs, SCEA subcutaneous injection of E-MenSCs in the abdomen, *SCEB* subcutaneous injection of E-MenSCs in the back.

The original Article has been corrected.

